# Comparative Assessment of Particulate Air Pollution Exposure from Municipal Solid Waste Incinerator Emissions

**DOI:** 10.1155/2013/560342

**Published:** 2013-07-14

**Authors:** Danielle C. Ashworth, Gary W. Fuller, Mireille B. Toledano, Anna Font, Paul Elliott, Anna L. Hansell, Kees de Hoogh

**Affiliations:** ^1^Department of Epidemiology and Biostatistics, MRC-PHE Centre for Environment and Health, Faculty of Medicine, Imperial College London, St Mary's Campus, Norfolk Place, London W2 1PG, UK; ^2^MRC-PHE Centre for Environment and Health, King's College London, Strand, London WC2R 2LS, UK; ^3^Small Area Health Statistics Unit, Department of Epidemiology and Biostatistics, MRC-PHE Centre for Environment and Health, Faculty of Medicine, Imperial College London, St Mary's Campus, Norfolk Place, London W2 1PG, UK

## Abstract

*Background.* Research to date on health effects associated with incineration has found limited evidence of health risks, but many previous studies have been constrained by poor exposure assessment. This paper provides a comparative assessment of atmospheric dispersion modelling and distance from source (a commonly used proxy for exposure) as exposure assessment methods for pollutants released from incinerators. *Methods.* Distance from source and the atmospheric dispersion model ADMS-Urban were used to characterise ambient exposures to particulates from two municipal solid waste incinerators (MSWIs) in the UK. Additionally an exploration of the sensitivity of the dispersion model simulations to input parameters was performed. *Results.* The model output indicated extremely low ground level concentrations of PM_10_, with maximum concentrations of <0.01 **μ**g/m^3^. Proximity and modelled PM_10_ concentrations for both MSWIs at postcode level were highly correlated when using continuous measures (Spearman correlation coefficients ~ 0.7) but showed poor agreement for categorical measures (deciles or quintiles, Cohen's kappa coefficients ≤ 0.5). *Conclusion.* To provide the most appropriate estimate of ambient exposure from MSWIs, it is essential that incinerator characteristics, magnitude of emissions, and surrounding meteorological and topographical conditions are considered. Reducing exposure misclassification is particularly important in environmental epidemiology to aid detection of low-level risks.

## 1. Introduction 

Incineration is being increasingly used as a waste management option in the United Kingdom (UK). This is in response to EU legislation restricting the amount of waste disposed of in landfills [1]. Up until the 1990s incineration in the UK was largely uncontrolled. Legislation pertaining to all incinerators in the UK, the EU Waste Incineration Directive (WID) (2000/76/EC), came into operation for new incinerators in 2002 and older ones in 2005. This has set strict limits on emissions into the air [2]; nonetheless, there remains public concern and scientific uncertainties about possible health risks from pollutants emitted from incinerators.

European waste legislation uses the Waste Hierarchy Framework to guide the use of different waste management options, prioritising the more environmental desirable and sustainable options. Incineration falls above disposal of waste in landfills within this framework but is not as desirable as recycling and composting, reuse, and prevention [3]. Municipal solid waste incinerators (MSWIs) burn waste assembled by collection authorities [4], at high temperatures, reducing the volume of waste, eliminating pathogens and are capable of recovering energy from the waste [5]. 

To date a number of epidemiological studies have investigated the relationship between incineration and health [4–12], with most focused on its association with risk of cancer and more recently, the risk of adverse birth outcomes [8, 12–24]. The UK Committee on Carcinogenicity of Chemicals in Food, Consumer Products and the Environment released a statement about MSWIs and cancer in 2000 (updated in 2009), stating that, “…*any potential risk of cancer due to residency near to municipal solid waste incinerators was exceedingly low and probably not measureable by the most modern epidemiological techniques*” [6, 7]. This was supported by the UK Health Protection Agency's' statement in 2009 “…*While it is not possible to rule out adverse health effects from modern, well regulated municipal incinerators with complete certainty, any potential damage to the health of those living close-by is likely to be very small, if detectable*” [25].However, the evidence base investigating this issue remains limited and most existing studies suffer from incomplete information on potential confounders, lack of statistical power, and poor exposure assessment.

Exposure assessment is often referred to as the “Achilles heel” of environmental epidemiology [26, 27]. Inaccurate and imprecise exposure estimates, leading to exposure misclassification, can create biases in health risk estimates. In many environmental epidemiology studies, exposure misclassification is unrelated to the health outcome, termed nondifferential exposure misclassification, which would be expected to bias observed effect estimates towards the null [28]. Accurate exposure assessment is particularly important for studies trying to detect/exclude small excesses in risk in relation to environmental exposures [29], such as due to incineration, in order to enable true risks, if present, to be detected. 

The methods used to assess exposure to an environmental source, such as an incinerator, range in design and complexity, from simple proxy methods to detailed individual level measures of exposure. Simple proxy methods, such as distance to the incinerator, assume a linear decrease in exposure with distance from source but benefit from the ease of implementation and the limited data and resources required to undertake a study using this exposure assessment method. However, this approach is crude and does not account for the magnitude of emissions, incinerator characteristics, or the propagation of the emissions due to local meteorological and topographic conditions. Individual level direct measures of exposure, such as biomarkers in human tissue, provide an objective assessment of exposure to chemicals and are considered “gold standards” in exposure assessment [30]. Biomarkers are often not feasible in large studies due to the high cost of laboratory analysis, the difficulties in acquiring human tissue, and the burden and potential risks to participants involved [30]. Exposure modelling has largely bridged the gap between the need for more accurate exposure assessment and the practical and financial constraints of large epidemiological studies. Atmospheric dispersion models use monitored emission data along with information on local topographic and meteorological conditions, within a Gaussian framework, to estimate the concentration and dispersion pattern of pollutants around an identified source [31, 32]. New generation dispersion models have an updated understanding of atmospheric turbulence and boundary layer structure [33] and have been extensively evaluated [34–37]. 

Many studies investigating the relationship between incineration and adverse health outcomes have used distance as a proxy for exposure. Some studies have included additional information alongside proximity to strengthen this method, including wind patterns, soil concentrations [18], local topography, and complaints of nuisance caused by the plumes [24]. Only a limited number of more recent studies have used dispersion models [8, 12, 13, 17, 23] to assess exposures. As far as the authors are aware, no existing studies on incinerators have compared these two exposure assessment methods and quantified the extent of exposure misclassification between the two. Modelled exposure patterns are expected to be different when using the two comparative methods. The distance method will predict greatest exposure adjacent to the stack and will decrease linearly with distance from the stack. These exposures will also be fixed in time and will be homogenous in space at a given distance from the stack. In contrast, because stack height above ground is considered, the dispersion model will predict low concentrations of incinerator emissions near to the stack. Greatest concentrations will be at a distance from the stack (determined by the release conditions and meteorology) after which concentrations will decrease nonlinearly with distance. Temporal changes in release conditions and meteorology are taken into account to produce a concentration field that varies in time. Here, we provide a detailed comparison of atmospheric dispersion modelling and a distance based method to assess exposure to particulates from two MSWIs and explore issues of exposure misclassification. 

## 2. Methods

### 2.1. Study Area and Study Population

Two UK MSWIs were included in this study, Crymlyn Burrows, located approximately 5 km east of Swansea, Wales and Marchwood, approximately 3 km west of Southampton, England. These two MSWIs are representative of operational MSWIs in Wales and England in terms of the operational standards they were built to (both have only ever operated to the most recent European Waste Incineration Directive [2]); their size (Crymlyn Burrows and Marchwood licensed throughput of 52,500 tonnes and 210,000 tonnes of MSW a year, respectively, where the typical median throughput of all operational UK MSWIs is 165,000 tonnes, ranging from 3,500 to 750,000 tonnes); and their rural locations (within 10 km surrounding Crymlyn Burrows 70% of the land is rural land and 69% for Marchwood, median for all operational MSWIs of 69%). The two selected incinerators additionally provided a number of contrasting features. Crymlyn Burrows has a single flue, is surrounded by hills, and lies 850 m from the coast, whereas Marchwood has two flues, is surrounded by flat land, and lies more inland. Incinerator characteristics and daily emissions data from their commissioning date (January 2003 for Crymlyn Burrows, January 2006 for Marchwood) until December 2010 were provided by the UK Environment Agency (EA).

The study area was defined as a 10 km radius around each MSWI. The 10 km distance was chosen for consistency with screening criteria used for implementing the Habitats Regulations: incineration plants that are within 10 km of a European Site require an assessment of their impact for short range air emissions. 

The study population was defined as all residents within the study area, calculated by extracting postcode headcount data from the 2001 census [38], where one UK postcode represents on average 12–15 properties and 40–45 people.

### 2.2. Emissions Dispersion Modelling

The Atmospheric Dispersion Modelling System Urban (ADMS-Urban) v2.3 modelling package was used [39] to model the dispersion pattern and ground level concentration of particles with a diameter <10 *μ*m (PM_10_) from both incinerators. ADMS-Urban is a new generation Gaussian plume air dispersion model that uses an updated understanding of turbulence and atmospheric boundary layer structure [33] and is capable of simulating the atmospheric dispersion patterns of pollutants from multiple sources and within complex terrain [40]. 

ADMS-Urban calculates atmospheric boundary layer parameters such as boundary layer height and Monin-Obukhov length from a variety of input parameters [40]: air temperature (°C), wind speed (m/s), wind direction (°), and cloud cover (oktas). The Monin-Obukhov length is an indicator of the atmospheric stability and is a key parameter in the dispersion of pollutant. It is defined by a quotient of heat flux at ground level by frictional velocity. It provides a height at which turbulent flows are created by buoyancy and not wind shear. In ADMS-Urban a minimum value for the Monin-Obkhov length is set, with the default value set to 30 m in order to account for the heat island effect of major cities and to prevent the model from stabilising [40, 41].

Another key model parameter that has impact on the dispersion of pollutants is the surface roughness length. Surface roughness length characterises the roughness of the terrain, providing an indicator of how much drag the wind experiences from the ground. Surface roughness is required to calculate convective turbulence.

#### 2.2.1. Model Input Data

For each MSWI, information on the location of the stack, year commissioned, total annual waste licensed to incinerate and stack characteristics was extracted from their environmental permit application to the EA. The precise location of the stacks was verified by checking the incinerator address and postcode against six-figure grid references (georeferenced location of the stack in British National Grid projection), in addition to visually searching for stacks on satellite maps in Google maps. Stack data included number of lines, stack height (m), stack diameter (m), exit velocity (m/s), exit flow (m^3^/s), and exit temperature (°C) (Table 1). For Marchwood only one measure of flue gas flow, velocity, and temperature was available from 2006 till 2010. For Crymlyn Burrows quarterly measures of these flue gas metrics were available for most years of operation. Annual averages of these quarterly measures were calculated and used. When quarterly measures were unavailable, the overall representative flue gas measures for Crymlyn Burrows were used. The concentration of total particulates at the flue exit for each MSWI was measured as daily means.

**Figure 1:**
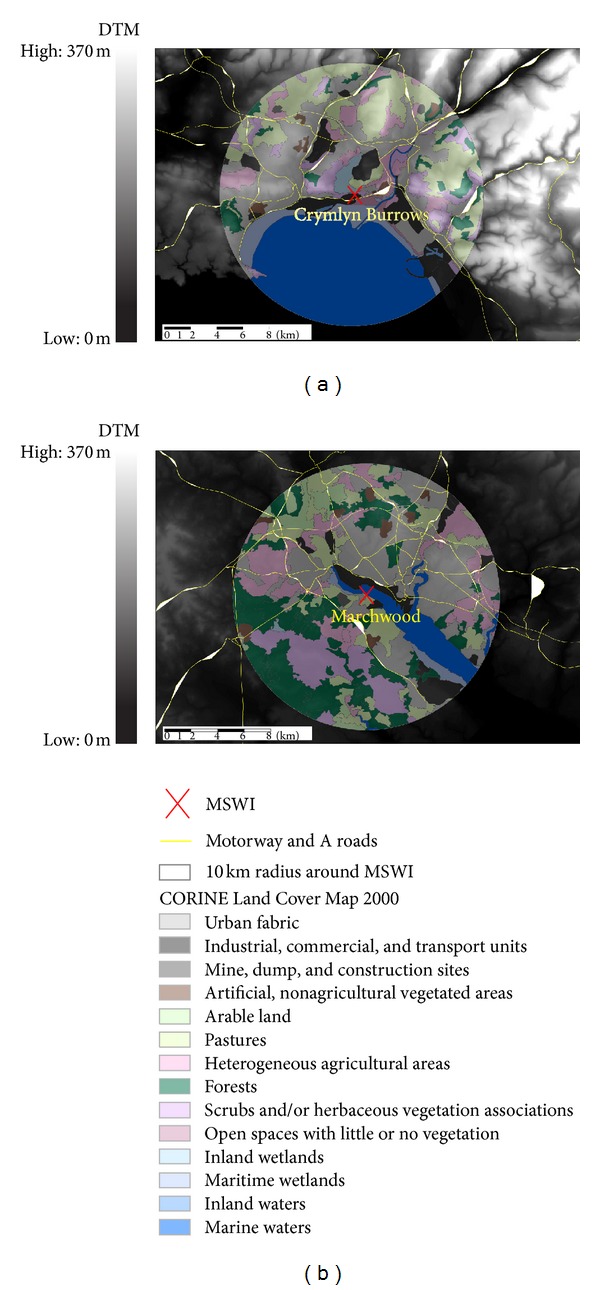
Land cover data from CORINE-Land 2001 and topography data from PANORMA 10 km around Crymlyn Burrows (a) and Marchwood (b) incinerators.

Sensitivity analysis of the dispersion conditions was conducted to select the most appropriate and representative surface roughness and Monin-Obukhov lengths. The fetch for roughness is defined by the US Environmental Protection Agency (US EPA) as 1 km surrounding the source [42]. Land cover data, extracted from the CORINE Land Cover Map 2000 [43] (Figure 1), was used to characterise the 1 km area around each MSWI. CORINE is an EU-wide dataset, generated by semiautomatic classification of satellite imagery [43] and comprises 44 land cover classes, of which 11 relate to urban land. Based on the land cover data around each MSWI, an array of relevant lengths was selected. As both MSWIs were partly surrounded by urban land cover (Marchwood 20% and Crymlyn Burrows 26%, resp., see Figure 1), a number of different surface roughness lengths and minimum Monin-Obukhov lengths were explored. Output concentrations were then compared when using the different values for both lengths.

The surface elevation in the area surrounding the MSWIs was extracted from Ordnance Survey PANORAMA digital terrain model (DTM), which has a horizontal resolution of 50 m [44]. As shown in Figure 1 the terrain surrounding Marchwood is low lying with a mean elevation of 23 m above sea level. However for Crymlyn Burrows there is a significant variation in elevation, with a range of 370 m. In order to account for this variation in terrain and therefore changes in the dispersion pattern of particulates, the hill option in ADMS-Urban was selected and a preprepared terrain file was extracted from the DTM and input into the model.

Meteorological conditions greatly influence the observed spatial pattern of emitted pollutants from a point source. Selecting an appropriate meteorological station, that best represents the area surrounding the MSWI, is therefore crucial. Hourly land surface meteorological observations from all Met Office stations in England and Wales between 2003 and 2010 were obtained from the British Atmospheric Data Centre (BADC). Candidate meteorological stations located within approximately 30 km from the selected MSWIs were identified. Meteorological stations considered were those with 90% completeness for all weather variables (excluding cloud cover), for each year. The Air Quality Modelling Assessment Unit (AQMAU) at the EA advised that incorporating cloud cover from alternative nearby stations makes a very small contribution to overall modelling uncertainties. Therefore, cloud cover was obtained from the nearest station with 90% completeness where necessary [45]. Following the selection of candidate meteorological stations, wind roses were plotted for each station. These wind roses were used to spot anomalies in the data (e.g., apparent gaps in wind from a given sector) and comparisons were made between the sites. Following this, CORINE land cover and DTM data were extracted and compared for a 1 km radius around each meteorological station in order to select a meteorological station with similar surrounding topography and land use to the MSWI. The dispersion model was then run using these different meteorological stations and their outputs compared. 

#### 2.2.2. Model Output

Bag-filtered stack emissions from the MSWIs were not considered to contain a significant amount of particulates greater than 10 *μ*m diameter. Emitted particulates were therefore modelled as PM_10_ and considered to disperse in the same manner as a gas.

Modelled ground level concentrations of PM_10_ for the sensitivity analysis were estimated for receptors in a 200 m × 200 m grid within the study areas. For Marchwood sensitivity analysis was performed for 2006 and Crymlyn Burrows for 2003. 

For the exposure analysis, all residential postcode centroids within the study area were used as receptors and ground level concentrations of PM_10_ were modelled. For Marchwood models were run for 2006–2010 and Crymlyn Burrows 2003, 2005–2010.

For the exposure analysis, each modelled day required input of single daily mean particulate concentrations at the flue exit together with hourly meteorological data to produce a daily ground level PM_10_ concentration field. These daily modelled concentrations were aggregated to calculate annual means. Model outputs were mapped in ESRI ArcMap 10.0 [46].

### 2.3. Distance to Source

All residential postcode centroids within the study area were assigned a distance to their respective MSWI using the NEAR function in ArcGIS. The distance metric was chosen as distance from the edge of the study area rather than distance from the incinerator. This was termed proximity and had its greatest value at the incinerator and least value at the edge of the study area. The ordering of the magnitude of the proximity metric allowed a clearer comparison of the distance and dispersion approaches with the greatest proximity value and highest concentration found closest to the incinerator.

### 2.4. Comparison of Exposure Assessment Methods

All residential postcodes within the study areas were assigned both an average modelled PM_10_ concentration over the period in which the MSWI was in operation and a distance to the MSWI. Postcodes were classified into deciles, quintiles, and tertiles from high to low exposures (modelled PM_10_ concentrations sorted from high to low, distance to MSWI from low to high). A population was additionally assigned to each postcode using headcount data extracted from the 2001 census [38].

The comparison of exposure assessment by the dispersion model and by the distance method was undertaken in three ways.

1. Calculation of Cohen's kappa coefficients of agreement between exposure deciles, quintiles, and tertiles as calculated by the distance method versus the dispersion model. Cohen's kappa coefficient provides a statistical measure of interobserver agreement taking into account chance, that is, a quantification of precision [47, 48]. Kappa coefficients range from 0 to 1, with 0 indicating no agreement and 1 perfect agreement between methods. As our exposure tertiles, quintiles, and deciles are ordinal categories, equal weighted kappa coefficients were calculated in addition to unweighted Cohen's kappa coefficients [49]. Weighted Cohen's kappa coefficients account for ordinal differences in categories; that is, a difference of two categories between the indices of exposure is a more severe misclassification error than a difference of one category.
2. Calculation of weighted and unweighted Cohen's kappa coefficients of agreement between the distance method and the modelled particulate concentrations by population weighted exposure deciles, quintiles, and tertiles.
3. Plotting of modelled long-term average PM_10_ concentrations against distance from the MSWI at each postcode centroid, with calculation of Spearman's correlation coefficients.

## 3. Results

### 3.1. Particulate Emissions from MSWI

**Figure 2:**
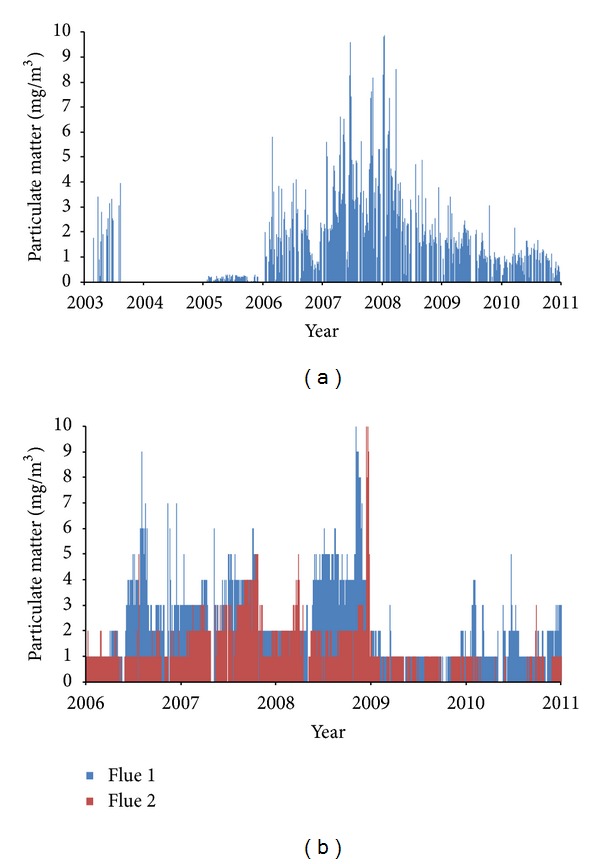
(a) Daily particulate concentrations measured at flue exit for Crymlyn Burrows from 2003 to 2010. (b) Daily particulate concentrations measured at flue exit for Marchwood from 2006 to 2010.

Figures 2(a) and 2(b) display the daily concentrations of total particulates measured at the flue exit for Crymlyn Burrows and Marchwood, respectively.  Figure 2(a) demonstrates the variability in concentrations for Crymlyn Burrows over the study period, 2003–2010, with the maximum concentration of 9.87 mg/m^3^. The gap in the data shown for 2004 was due to a fire during the last quarter of 2003 causing Crymlyn Burrows to stop operation during 2004.  Figure 2(b) shows the daily particulate concentrations for both flues for the Marchwood incinerator. Again, there was considerable variability in concentrations over time and also between the two flues. Both Flue 1 and 2 had a maximum concentration of 10 mg/m^3^, the Waste Incineration Directive limit. Both MSWIs show a decreasing trend in particulate emissions from 2008 (Crymlyn Burrows) and 2009 (Marchwood) until 2010, from daily emissions of ~10 mg/m^3^ to 1-2 mg/m^3^. The maximum particulate emissions took place in 2008 for both MSWIs.

### 3.2. Dispersion Modelling

**Figure 3:**
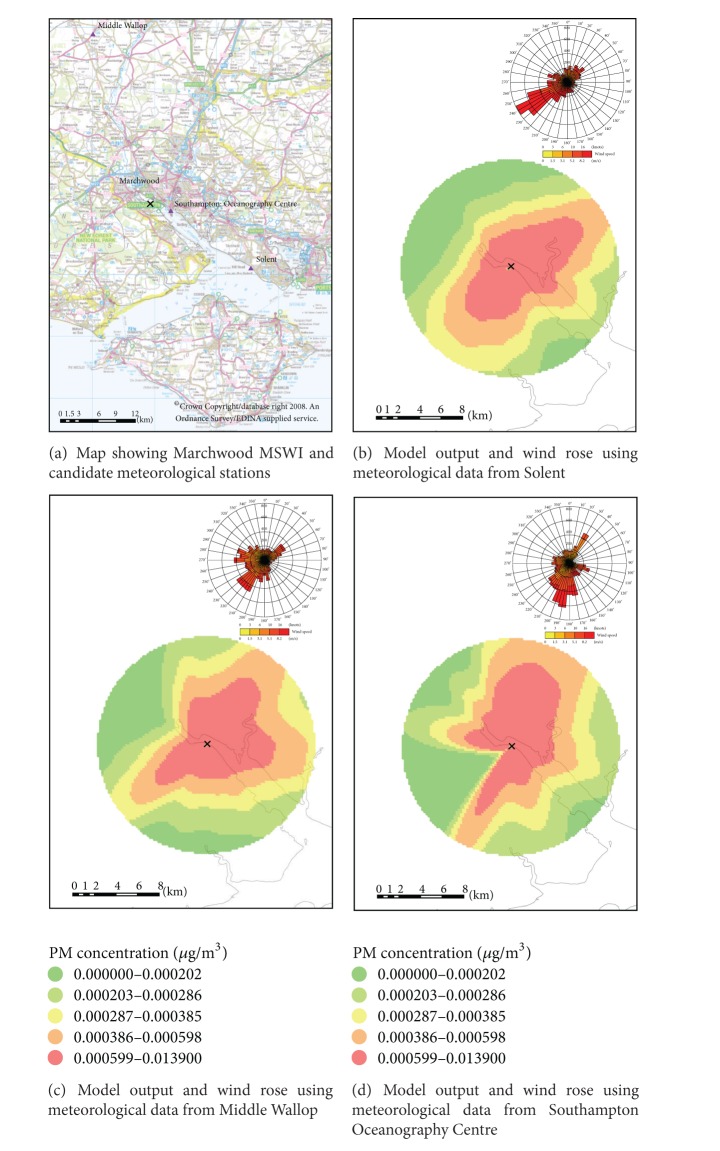
Sensitivity of the model to the selected meteorological stations for Marchwood in 2006. Maps (b)–(d) use the same site surface roughness length and minimum Monin-Obukhov length.

For Marchwood, three candidate meteorological stations were located within 30 km. The nearest meteorological station was Southhampton Oceanography Centre located 3.3 km east of Marchwood, followed by Solent (19.1 km south-east) and Middle Wallop (29.2 km north) (see Figure 3). For Crymlyn Burrows only one meteorological station was available located 9.4 km south-west from the incinerator. 

Comparisons were made between the three meteorological stations available for Marchwood. First, the wind roses for the three meteorological stations were compared. The wind rose for the Southhampton Oceanography Centre displayed very low frequency of wind from the north-east, between 50 and 80 degrees, for all years of operation (2006–2010) (Figure 3(d)). The other two meteorological stations, however, did not show this pattern (Figures 3(b) and 3(c)). The effect of this apparent gap in wind direction becomes particularly evident when using the data from these meteorological stations in our dispersion model simulations. Figure 3 shows the modelled annual mean particulate concentrations in 2006 using the three meteorological stations around Marchwood MSWI. The PM_10_ annual mean concentration using the Southampton Oceanographic Centre clearly shows a gap in the predicted concentrations south-west of the incinerator (Figure 3(d)), not seen when using the other two meteorological stations (Figures 3(b) and 3(c)). Based on this comparison the data from Southampton Oceanographic Centre meteorological station was deemed erroneous for unknown reasons and was therefore not used in subsequent analysis. The wind and dispersion patterns were similar for Solent and Middle Wallop, with higher PM_10_ concentrations in the SW-NE diagonal. Therefore the closest station, Solent, was selected for the exposure analysis. However when Solent cloud cover fell short of 90% capture annually, cloud cover data from Middle Wallop was used.

**Figure 4:**
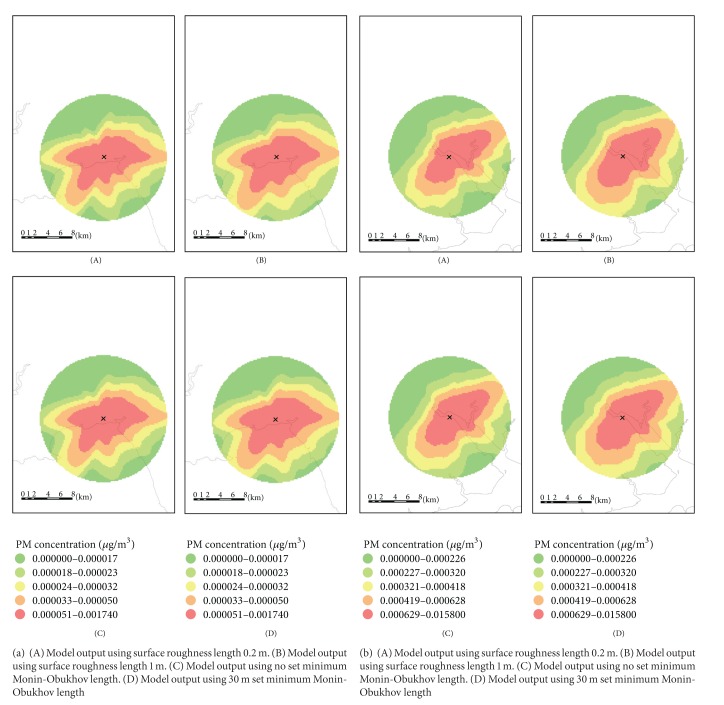
(a) Sensitivity of the model to site surface roughness length and minimum Monin-Obukhov length for Crymlyn Burrows. Maps (A)–(D) use the same meteorological station data for 2003. (b) Sensitivity of the model to site surface roughness length and minimum Monin-Obukhov length for Marchwood. Maps (A)–(D) use the same meteorological station data for 2006.

An exploration of surface roughness for both MSWIs showed little variation in the model output for surface roughness lengths varying from 0.2 m to 1 m (see Figures 4(a) and 4(b)). Only 7.7% of the model receptors had a difference in modelled particulate concentrations greater than 25% in Marchwood and 3.1% for Crymlyn Burrows (Table 2). The difference in modelled particulate patterns and concentrations between no set minimum Monin-Obukhov length and 30m showed little variation, with a maximum percentage difference of 31% for Marchwood and 18% for Crymlyn Burrows (Table 2). 

**Figure 5:**
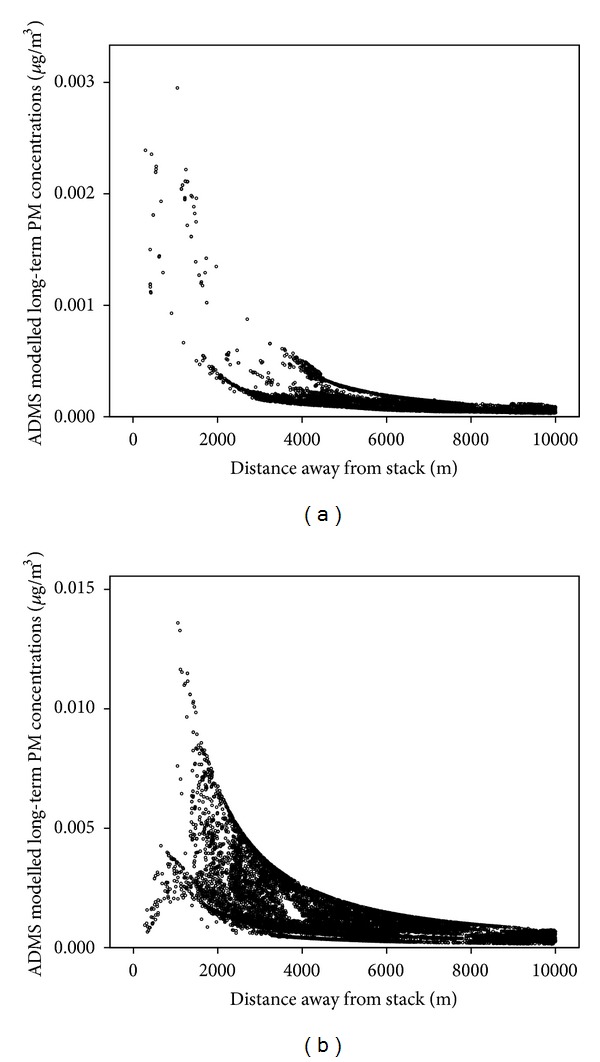
(a) Modelled long-term PM concentrations (*μ*g/m^3^) plotted against distance away from the MSWI (m) at postcode centroid for Crymlyn Burrows. (b) Modelled long-term PM concentrations (*μ*g/m^3^) plotted against distance away from the MSWI (m) at postcode centroid for Marchwood.

Table 3 demonstrates the extremely low concentrations of modelled annual PM_10_ concentrations within 10 km from the MSWI both for all days of the year (Table 3(a)) and also for only the days of operation (Table 3(b)). The modelled ground level concentrations of PM_10_ were extremely low for both MSWIs, with a mean concentration of 0.000117 *μ*g/m^3^ for Crymlyn Burrows for all days and 0.000334 *μ*g/m^3^ for operational days only; and 0.00129 *μ*g/m^3^ for Marchwood for all days and 0.00205 *μ*g/m^3^ for operational days only. Modelled long-term average PM_10_ concentrations were very small (maximum of 0.0022 *μ*g/m^3^ for Crymlyn Burrows and 0.0089 *μ*g/m^3^ for Marchwood).  Figure 5 shows the modelled long-term average PM_10_ concentrations for both MSWIs against distance from the MSWI at each postcode centroid. It is clear from Figure 5 that the concentrations at the 10 km edge of the modelled domain were <7% of the maximum concentration. 

The pattern of the final dispersion model for Crymlyn Burrows showed irregular shapes, with symmetrical bands of increasing exposure from the source. This irregular dispersion pattern might be due to the hilly topography in the Swansea area that modifies the wind patterns and, therefore, the dispersion of emissions from the MSWI. Due to its coastal location a large proportion of the modelled area has no population. For Marchwood the final dispersion pattern was much more elliptical with the greatest PM_10_ concentration extending to the north-east of the MSWI following the main wind direction.

### 3.3. Distance to Source

Table 4 shows the number of postcodes and the population count in relation to distance from the two MSWIs. The area around the Marchwood MSWI is more densely populated (361,005 people within 10 km) than Crymlyn Burrows (248,937 people within 10 km). The population around Marchwood MSWI also resides closer to the MSWI than that at Crymlyn Burrows with the greatest population density between 3 km and 7 km. 

### 3.4. Comparison of Exposure Assessment Methods

The agreement between exposure categories, as calculated by the dispersion modelling and distance methods, is shown in Table 5. Better agreement was achieved when using tertiles (Cohen's kappa coefficient of 0.424 unweighted and 0.553 weighted and 0.308 unweighted and 0.448 weighted from Crymlyn Burrows and Marchwood, resp.) compared with deciles and quintiles (Cohen's kappa coefficient ranging from 0.068 to 0.201 unweighted and 0.198 to 0.519 weighted).

Table 6 shows the population weighted agreement of the two exposure methods. Again, agreement improved with a reduction in the numbers of exposure categories. Best agreement between methods was displayed for Crymlyn Burrows exposure tertiles (but here the unweighted Cohen's kappa coefficient only reached 0.425, equally weighted Cohen's kappa coefficient only reached 0.548) and the poorest agreement for Marchwood exposure deciles (unweighted Cohen's kappa coefficient 0.0644, equally weighted Cohen's kappa coefficient 0.150).

Figure 5 shows the long-term mean PM_10_ concentration at each postcode centroid against distance from MSWI for Crymlyn Burrows (Figure 5(a)) and Marchwood (Figure 5(b)). Spearman correlations (*R*) for modelled long-term PM_10_ concentrations versus proximity from the edge of the modelling domain at postcode level were 0.765 and 0.688 for Crymlyn Burrows and Marchwood, respectively (both significant at the 0.01 level). 

## 4. Discussion 

The majority of the studies exploring the relationship between incineration and health have used a simple distance metric as a proxy for exposure. Here we have provided a comparison of distance from source and emissions modelling to assess exposure to particulates emitted by two MSWIs in the UK. Our results suggest that epidemiological studies requiring an assessment of exposure to airborne pollutants from MSWIs, at a small scale level, would benefit from a dispersion modelling approach compared to a simple distance based approach. Although the use of distance as a proxy for exposure has limited data requirements, it does not account for source characteristics, the concentrations of pollutants emitted, local meteorological conditions, and topography [31, 50] all of which are incorporated in Gaussian dispersion models, such as ADMS-Urban. Dispersion models provide a different exposure assessment to distance from source. This approach is expected to be more realistic than a simple distance proxy as it tries to capture the physical processes that determine the dispersion of emissions from a point source including topographic and meteorological information that influence where and how emissions are dispersed. ADMS-Urban has been successfully used and validated when assigning exposure at an individual or small area level [34–37] and is frequently used for regulatory purposes, policy support, and providing information to the public [32]. Dispersion modelling can additionally help determine the distance to which a particular source influences exposures, as shown in Figure 5, where modelled PM_10_ falls to <7% of peak concentrations at 1000 m to 2000 m away from the MSWI. The comparison between dispersion modelling and distance for the two MSWIs studied here (see Table 5 and Figure 5) reveals poor to moderate agreement only when using distance compared with dispersion modelling. Both methods assigned a decreasing exposure with an increasing distance from source (as shown by the strong spearman's correlations with continuous measures). However, when using categorical metrics (as are often employed in epidemiological studies) distance was a fairly good proxy in distinguishing highest and lowest exposure tertiles, but the dispersion model was able to capture the pattern of small area level variation in population exposure (Figures 3 and 5), which did not conform to circular dispersion around the source as would be predicted using a distance model.

The influence of stack height on the dispersion pattern was especially apparent for the Marchwood MSWI, which shows very small PM_10_ concentrations up to approximately 500 m (Figure 5(b)), after which they peak between 1000 and 2000 m, depending on the direction. This pattern was less apparent at the Crymlyn Burrows MSWI, mainly due to the lack of postcodes within 2000 m of the MSWI. 

Both Figures 5(a) and 5(b) show a flattening in modelled PM_10_ concentrations beyond approximately 5 km, suggesting that, at least for the Crymlyn Burrows and Marchwood MSWI, most variability in exposure occurs within 5 km of a MSWI and this was therefore captured well within the 10 km distance chosen in this assessment.

Model input parameters influenced both the pattern and concentration of the modelled PM_10_, in turn affecting the modelled exposed population. It is therefore essential that the quality of the model input parameters is assessed. It was found that the model was sensitive to surface roughness length, Monin-Obukhov length, and meteorological conditions. The model output showed little relative variation in output concentrations with different input parameters with the exception of changes in meteorological station. We demonstrate here that the choice of meteorological input data is crucial. As shown in Figure 3, possible misclassification of exposure is evident from the use of different meteorological stations, particularly in the case of the south-west part of the Marchwood MSWI.

The dispersion model simulations in this study were subject to a number of limitations that would contribute to the uncertainty in the ground level exposure estimates produced. Firstly, Marchwood only had a single measure of flue gas flow, velocity, and temperature for the duration of its operation (2006 till 2010), whereas Crymlyn Burrows had quarterly measures of these flue gas metrics for most years of operation which showed substantial variation. The assumption that these flue exit parameters are constant over such long periods of time is therefore not representative of true conditions. Additionally, due to data availability, poor data quality, and completeness, the choice of meteorological sites was limited and it was challenging to find meteorological sites representative of the surrounding area. This was especially evident for Marchwood where the selected meteorological site (Solent) was located 19 km away from the MSWI. Additionally, although ADMS-Urban has been validated as a point source modelling tool in other scenarios, the long-term mean concentrations of modelled PM_10_ in this study were exceptionally low, and therefore model validation would not be possible, as they fall below the limit of detection for regulatory ambient measurements.

There are a number of disadvantages to using dispersion models, including their large input data demands, which are often unavailable, and the expertise required to successfully run and interpret the models [31]. To meet the EU Directive requirements the MSWIs in this study, along with those elsewhere in Europe, are now required to have daily measurements of particulate emissions. This allows time varying emissions to be included in modelled assessments for the first time. This is beneficial for calculating exposures linked to health endpoints with critical exposure periods, for example, trimester specific exposures for birth outcomes. 

Although long-term ground level PM_10_ levels from these MSWIs were found to be approximately thousandths of regional background levels, it is hypothesised that particulates from MSWIs may possibly have different impacts on health than those from other ambient sources of particulate matter due to their metal or dioxin content, for instance. The modelled concentrations of PM_10_ may act as a proxy for the concentration fields for these and other primary emissions from MSWIs. While long-term PM_10_ concentrations from dispersion modelling may provide a good indication of ambient concentrations, this will still be an imperfect marker of personal exposure. An alternative individual level exposure could be measured by personal monitoring or collection and analysis of biomarkers. However, such personal exposure approaches, aside from being very expensive and time-consuming and (for biomarkers) potentially invasive, may not adequately capture exposures specific to MSWIs. 

## 5. Conclusions

Using distance as a proxy measure of exposure to emissions from incinerators is a simple, quick, and cheap approach; however, when compared with dispersion modelling, there is indication of exposure misclassification. Dispersion models incorporate information on individual incinerator characteristics, emission concentrations, local meteorological conditions, and topography, all of which contribute to the observed concentrations and spatial patterns of incinerator emissions. The additional detail included in these models enables a more appropriate and informative exposure assessment from incinerators, which is important in an epidemiological context in order to reduce risk of bias in risk estimates due to exposure misclassification. 

## Figures and Tables

**Table 1:** Source characteristics of the two inclusive municipal solid waste incinerators.

Incinerator	County	Permitted throughput (tonnes/year)	Flue	Stack height (m)	Stack diameter (m)	Flue exit flow rate (m^3^/s)	Flue exit velocity (m/s)	Temperature (°C)
Crymlyn Burrows	Neath Port Talbot (South Wales)	52,500	1	40	0.95	12.3	17.6	136
Marchwood	Hampshire (England)	210,000	1 2	65 65	1.25 1.25	30.3 30.9	24.7 25.2	150 148

Flue exit flow rate, velocity, and temperature for Crymlyn Burrows provided are a mean of biannual measurements for most years of operation, whereas for Marchwood these measures are single measures derived from the permit application.

**Table 2:** Surface roughness sensitivity analysis. Percentage difference between extreme surface roughness values at all model receptors.

Percentage difference	Crymlyn Burrows	Marchwood
Surface roughness		
Mean (%)	8.7	12.3
Median (%)	6.9	11.2
Minimum (%)	0	0
Maximum (%)	116.6	117.5
Receptors > 25% difference (%)	3.1	7.7
Monin-Obukhov length		
Mean (%)	6.4	11.5
Median (%)	5.5	10.2
Minimum (%)	0	0
Maximum (%)	17.6	30.6
Receptors > 25% difference (%)	0	6.6

**Table:** (a)

	Mean (×10^−5^ *μ*g/m³)	Median (×10^−5^ *μ*g/m³)	Interquartile range (×10^−5^ *μ*g/m³)
Crymlyn Burrows			
2003	3.7	3.0	2.5
2005	0.8	0.6	0.5
2006	10.8	8.2	6.8
2007	29.4	24.2	17.1
2008	22.0	16.5	15.2
2009	10.3	7.7	7.1
2010	4.9	3.9	3.0
Marchwood			
2006	121.5	80.0	101.6
2007	186.3	127.7	149.9
2008	229.5	139.5	200.8
2009	59.6	44.5	50.3
2010	48.9	36.4	32.2

**Table:** (b)

	Mean (×10^−5^ *μ*g/m³)	Median (×10^−5^ *μ*g/m³)	Interquartile range (×10^−5^ *μ*g/m³)	Days of operation		
Crymlyn Burrows						
2003	51.5	4.25	31.7	33		
2005	2.61	2.10	1.59	150		
2006	29.1	23.6	17.1	204		
2007	61.2	51.1	33.6	264		
2008	52.7	42.6	32.6	227		
2009	23.1	18.8	13.8	225		
2010	13.3	10.6	7.77	188		

				Flue 1	Flue 2	Either one or both flues in operation

Marchwood						
2006	207.3	140.9	154.3	308	240	334
2007	290.6	204.1	221.5	325	327	344
2008	338.1	212.7	262.7	340	325	358
2009	96.9	72.5	72.0	296	192	357
2010	91.7	69.8	59.1	323	104	356

**Table 4:** Distance of the study population (all residents within 10 km) to the incinerators, Crymlyn Burrows and Marchwood.

Distance to source (km)	Crymlyn Burrow				Marchwood			
	Number of PCs	Percentage of total PCs (%)	Population count	Percentage of total population (%)	Number of PCs	Percentage of total PCs (%)	Population count	Percentage of total population (%)
0–<1	22	0.2	165	0.1	87	0.5	1677	0.5
1–<2	69	0.5	834	0.3	813	4.2	12829	3.6
2–<3	229	1.8	5067	2.0	2363	12.3	31729	8.8
3–<4	777	5.9	14590	5.9	2720	14.2	46690	12.9
4–<5	2623	20.1	38736	15.6	2969	15.5	59070	16.4
5–<6	2496	19.1	49338	19.8	2999	15.6	69784	19.3
6–<7	2365	18.1	51665	20.8	2171	11.3	48832	13.5
7–<8	1982	15.2	39467	15.9	1378	7.2	25298	7.0
8–<9	1256	9.6	25853	10.4	1611	8.4	32654	9.0
9–10	1251	9.6	23222	9.3	2055	10.7	32442	9.0

Total	13070	100	248937	100	19166	100	361005	100

PC: postcodes.

**Table 5:** Measure of agreement Kappa coefficient (where 0 = no agreement; 1 = perfect agreement) between modelled long-term PM_10_ concentrations and distance away from stack categorised in deciles, quintiles, and tertiles at postcode level.

	*N*	Type of Kappa	Deciles	Quintiles	Tertiles
Crymlyn Burrows	13069	Unweighted	0.0684	0.210	0.424
		Weighted-Equal	0.307	0.519	0.553
Marchwood	19166	Unweighted	0.0734	0.177	0.308
		Weighted-Equal	0.198	0.446	0.448

**Table 6:** Measure of agreement Kappa coefficient (where 0 = no agreement; 1 = perfect agreement) between population weighted modelled long-term PM_10_ concentrations and distance from the stack categorised in deciles, quintiles, and tertiles at postcode level.

	*N*	Type of Kappa	Deciles	Quintiles	Tertiles
Crymlyn Burrows	5269	Unweighted	0.0932	0.251	0.425
		Weighted-Equal	0.334	0.535	0.548
Marchwood	8102	Unweighted	0.0644	0.169	0.219
		Weighted-Equal	0.150	0.380	0.345
